# Differential energy partitioning occurs in response to high- and low-energy diets in Holstein cows during late lactation

**DOI:** 10.3168/jdsc.2025-0891

**Published:** 2026-01-16

**Authors:** Thiago O. Cunha, Tanya L. France, Sebastian I. Arriola Apelo, Kenneth.F. Kalscheur, Elizabeth A. French, Mateus Z. Toledo, Milo C. Wiltbank, Laura L. Hernandez

**Affiliations:** 1 Department of Animal & Dairy Sciences, University of Wisconsin–Madison, Madison, WI 53706; 2 US Dairy Forage Research Center, USDA-Agricultural Research Service, Madison, WI 53706; 3 Purina Animal Nutrition, Land O'Lakes Inc., Sun Prairie, WI 53718

## Abstract

This study evaluated the impact of high-energy (HE) or low-energy (LE) diets during late lactation and the responsiveness of body condition changes to diet during the dietary treatment period. Sixty-six multiparous Holstein cows were blocked by parity and expected date of parturition and randomly assigned to HE (1.74 Mcal/kg DM) or LE (1.50 Mcal/kg DM) diets at 150 d of gestation to achieve high or average BCS by dry-off (233 d of gestation). Surprisingly, not all cows within dietary treatment groups were responsive to the diet based on BCS at dry-off. To determine the underlying physiology responsible for this observation, we classified cows as responsive (R) or nonresponsive to diet (NR) within each dietary treatment: HE (HE-R, >0.25 increase in BCS [28/35]; HE-NR, ≤0.25 BCS [7/35]) or LE (LE-R ≤0.25 increase in BCS [27/31]; LE-NR >0.25 BCS increase [4/31]). Feed intake did not differ within the HE-R and HE-NR subgroups or between the LE-R and LE-NR subgroups. During the dietary treatment period, cumulative ECM was greater in HE-NR than HE-R cows (3,304 vs. 2,236 ± 151 kg). In contrast, LE-NR produced less ECM than LE-R cows (1,622 ± 229 vs. 2,068 ± 77 kg) during the dietary treatment period. Circulating insulin concentrations were similar among subgroups at study enrollment (9.2 ± 1.1 mU/mL); however, dry-off insulin concentrations differed among subgroups (HE-R 17 ± 3.0; HE-NR 5.4 ± 2.0 mU/mL; LE-R 10.2 ± 2.0; LE-NR 23.6 ± 4.0 mU/mL). One week before parturition, circulating insulin concentrations converged and were no longer different among subgroups, averaging 12.5 ± 2 mU/mL. Our data suggest that circulating insulin concentration is involved in regulating the response to dietary treatment during late lactation.

## Highlights

• Insulin resistance mediates milk production to control body condition in late lactation.
• High-energy-fed cows that did not gain body condition increased milk production.
• Low-energy-fed cows that gained body condition decreased milk production.
• High-energy responsive and low-energy nonresponsive cows had the highest insulin concentrations.

## Graphical Abstract

**Figure:**
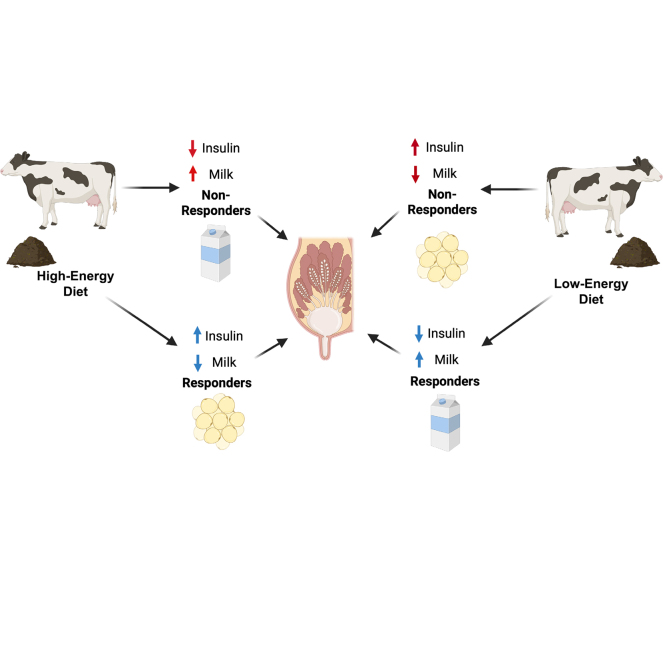
**Summary:** Cows fed high- and low-energy diets differentially exhibited changes in body condition by altering milk production and circulating insulin concentrations. Cows fed a low-energy diet that did not alter their body condition during the late-lactation treatment period had decreased milk production and increased insulin concentrations compared with responsive cows. Cows fed a high-energy diet that did not increase their body condition during the late-lactation treatment period had increased milk production and reduced insulin concentrations compared with the responsive cows. Figure created in BioRender.

In dairy cattle, homeorhetic regulation represents a fundamental physiological adaptation, and it is particularly evident in high-producing dairy cows during the periparturient period. Cows often undergo a negative energy balance (**NEB**) phase as they adjust to the heightened demands of milk production over a condensed period. This NEB typically occurs due to an imbalance between the energy required for sustaining high levels of milk production and the energy provided by feed intake. This discrepancy often results in the mobilization of endogenous energy reserves, predominantly from adipose tissue, to meet increased metabolic demands by the mammary gland for milk synthesis (Roche et al., 2009).

During the early stages of lactation, mobilization of adipose tissue is crucial for supplying energy necessary to sustain elevated milk production (Drackley, 1999). As lactation advances, dairy cows enter a phase of positive energy balance, during which they can restore their body reserves in preparation for the next lactation cycle. This phase is critical for maintaining long-term health and productivity. Effective management of the magnitude and extent of positive energy balance is critical, as inadequate nutritional management can lead to over-conditioned states (Schuh et al., 2019; Ghaffari et al., 2023). Over conditioning is associated with a range of negative outcomes in subsequent lactations, including exacerbated NEB, compromised health during the early postpartum period, and diminished productive and reproductive performance (Barletta et al., 2017; Schuh et al., 2019).

Nutritional management is used to modulate energy responses in dairy cows at various stages of lactation (Maltz et al., 2013; Allen, 2023); however, individual cows do not always respond to different feeding regimens. Responses to the diets provided can vary greatly depending on herd factors, such as stocking rate and diet composition, or cow factors, including the age of the cow or genetic merit (Roche et al., 2009). Such variations indicate that the regulation of energy reserves is influenced by a complex interplay of factors, including net energy input and output, but also genetic predispositions.

The primary objective of this hypothesis-generating, post hoc study was to elucidate the characteristics of cows that either maintain or gain body reserves during periods of positive energy balance in late lactation. We tested the hypothesis that feeding Holstein cows high- or low-energy diets during late lactation would influence their BCS dynamics, insulin and glucose concentrations, and subsequent energy balance during the dry period and early lactation differentially at the cow-level.

This study was conducted at the US Dairy Forage Research Center (Prairie du Sac, WI) from October 2021 to November 2022. Cows were housed in tiestalls throughout the study, except during the early dry period, during which they were group-housed until close-up, when they were moved back to the tiestalls. Cows were milked thrice daily. All procedures involving animals were previously approved by the Institutional Animal Care and Use Committee of the University of Wisconsin–Madison under the protocol number A006319.

These data are observations made in part of a larger recently published study (Cunha et al., 2025). Briefly, 66 multiparous Holstein cows averaging 246 ± 3.7 DIM and 148 ± 0.5 d of gestation were studied. Cows were blocked by parity and expected calving date and then randomly assigned to one of 2 diets: low-energy (**LE**; 1.50 ± 0.01 Mcal/kg DM; n = 31) or high-energy (**HE**; 1.74 ± 0.01 Mcal/kg DM; n = 35). From dry-off onward (233 ± 3 d of gestation), all cows were offered the same ration formulated to meet their requirements during the dry period, close-up, and the subsequent lactation. Cows were milked 3 times a day, had free access to water, and fresh TMR was delivered once daily, with orts weighed the following day to calculate daily intake for each cow, aiming for a 5% to 10% refusal rate. We did not restrict feed intake. The mean values for each week were used to determine milk yield and feed intake.

To estimate body reserve changes, weekly measurements of BCS, backfat thickness (**BFT**), and BW were collected. Body condition score was measured as described by Edmundson et al. (1989), and BFT assessed as described by Schröder and Staufenbiel (2006). Body weight was obtained on 2 consecutive days immediately after morning milking, and the average between the 2 measurements was used for analysis.

Samples of TMR from both treatment diets, close-up, and new lactation were collected daily and mixed into a composite sample for each week of the study. Subsequently, weekly samples were mixed into a composite sample for each month. The dry period diet was sampled once a week. Full diet information is published in Cunha et al. (2025).

Milk yield was recorded at each milking by electronic milk flow meters, and the individual daily yield was calculated by adding the milk weight obtained from the 3 daily milkings. Milk samples were collected from 3 consecutive milkings in one day from each cow and were sent to a commercial laboratory (AgSource Milk Analysis Laboratory, Verona, WI) once a week for determination of composition of fat, true protein, lactose, and fatty acids (de novo, preformed, mixed).

Blood samples from coccygeal vessels were collected during morning periods, before or within 1 h after feeding. After sampling, blood tubes (10-mL BD Vacutainer, lithium heparin 158 USP units, 367880, Becton Dickinson), were kept refrigerated for ∼6 h, then centrifuged for 20 min at 2,500 × *g* at 4°C, and the was plasma harvested and stored at −80°C.

All calculations were performed using the mean values for each week. Energy content of milk was calculated following (NASEM, 2021), as follows: Milk NEL (Mcal/kg milk) = 9.29 × kg fat/kg milk + 5.85 × kg true protein/kg milk + 3.95 × kg lactose/kg milk. Energy-corrected milk for each cow was calculated as 0.3246 × milk yield (kg/d) + 12.86 × fat yield (kg/d) + 7.04 × protein yield (kg/d; NRC, 2001). Energy for maintenance (NASEM, 2021) was calculated based on metabolic BW (BW^0.75^), as follows: maintenance NEL (Mcal/d) = BW^0.75^ × 0.10. Estimates of energy requirement for gestation were based on (NASEM, 2021), using a calf with 44 kg BW at birth. Energy balance (**EB**) during late lactation period was defined as follows: EB (Mcal/d) = [intake NEL − (maintenance NEL + pregnancy NEL + milk production NEL)].

Plasma insulin concentrations were measured using a commercial ELISA (Bovine Insulin ELISA, Mercodia; Caputo Oliveira et al., 2019). The intra-assay CV was 9.8% and the interassay CV was 9.5%. Glucose concentrations in serum were analyzed by colorimetric plate assay (Autokit Glucose, Wako; Pszczolkowski et al., 2023). The intra-assay CV was 6.94% and the interassay CV was 12.5%. Glucose and insulin values were used to calculate the homeostatic model assessment of insulin resistance (**HOMA-IR**; Matthews et al., 1985; Hackbart et al., 2013).

Cows were categorized as responsive or nonresponsive to dietary treatment based on the BCS change at study enrollment versus the BCS at the week of dry-off. Cows in the HE group that gained ≥0.25 of BCS were considered as responsive to dietary treatment. In contrast, cows in the LE group classified as responsive to dietary treatment were those cows that lost, maintained, or gained up to 0.25 of BCS during the same period. Conversely, nonresponsive cows in the HE group gained ≤0.25 of BCS, whereas nonresponsive cows in the LE group gained more than 0.25 of BCS during the same period.

The sample size determination was initially based on achieving a difference of 0.5 in BCS between the groups at dry-off (3.25 vs. 3.75; Cunha et al., 2025). This study was based on observations of cows that did not respond as intended in the original study and was therefore exploratory in nature. This was a convenience sample; therefore, an a priori sample size calculation was not performed.

All statistical analyses in this study were performed using SAS (version 9.4, SAS Institute Inc.). Cow was considered the experimental unit. Mean, SD, and SEM were obtained using the MEANS procedure.

After enrollment, the analysis was divided into 3 main time periods: late lactation, dry period, and new lactation. Because parturition occurred on a range of days of gestation, data at the end of the dry period were normalized for weeks or days relative to parturition. In addition, data within the first 21 d of the new lactation period were analyzed separately, aiming to better characterize the periparturient period (Drackley, 1999).

Repeated measures variables such as BCS, BFT, BW, milk production, feed intake, or insulin concentrations over time (week relative to dry-off or relative to parturition) were analyzed using a generalized linear mixed model applying the MIXED procedure with the REPEATED statement with cow specified in the SUBJECT option of SAS. During the treatment phase and dry period, the model included the fixed effects of treatment, week, lactation (2 vs. 3+), and the interaction between week and treatment. Normality and homoscedasticity of residuals were assessed by visual inspection of studentized residual plots for each variable after fitting the model using the residual option of the MIXED procedure. The Kenward–Roger method was used to calculate the approximate denominator degrees of freedom for the *F*-tests in the statistical models. For the MIXED procedure, fit statistic parameters for unstructured, Toeplitz, compound symmetry, first-order autoregression, heterogeneous compound symmetry, and heterogeneous first-order autoregression variance structure were tested. The covariance structure with the lowest Akaike information criterion values was used for the analysis. When an interaction of treatment with time was detected as significant, treatment means at time points of measure were partitioned using the SLICE command of SAS. All analyses used a 2-tailed test. Probability values were considered different when *P*-values were ≤0.05 and considered a tendency toward difference when *P*-values were >0.05 and ≤0.10.

During the dietary treatment phase, 1 cow from the LE group aborted at 230 d of gestation. Two cows in the HE group aborted at 200 and 220 d of gestation. All cows were included in the dataset for the dietary treatment period analysis and then excluded from the dry period and postpartum analysis. Additionally, 2 cows from the HE group aborted during the dry period and were excluded from the dry period and the postpartum analysis.

Body condition score was different in HE and LE cows on the day of dry-off compared with the day of study enrollment (*P* < 0.001). In the HE group, 7/35 cows did not gain more than 0.25 points of BCS during the dietary treatment period, whereas 4/31 cows in the LE group gained more than 0.25 points, indicating that BCS did not change in the expected manner to the dietary treatment and designating them, by our definition, as nonresponsive (Figure 1A–B). Subsequently, cows were classified as HE responsive (**HE-R**), HE nonresponsive (**HE-NR**), LE responsive (**LE-R**), and LE nonresponsive (**LE-NR**) to dietary treatment during these late-lactation nutritional treatment groups. The BFT at dry-off was 45.8 and 36.8 ± 1.9 mm in the HE-R and HE-NR categories (*P* < 0.001; Table 1), respectively, whereas LE-R and LE-NR cows averaged 37.9 ± 1.0 and 41.3 ± 1.8 mm (*P* = 0.01; Table 1). Energy-corrected milk differed between BCS subgroups within dietary treatments (*P* < 0.01; Figure 1C). During the dietary treatment period, cumulative ECM production was greater for HE-NR than HE-R (3,304 vs. 2,236 ± 151 kg, respectively), and LE-NR produced less ECM than LE-R (1,622 ± 229 vs. 2,068 ± 77 kg, respectively). During the last week before dry-off, HE-R produced less ECM than HE-NR cows, whereas LE-R produced more ECM than LE-NR cows (*P* < 0.001; Table 1). Feed intake did not differ within each responsive versus nonresponsive group across dietary treatments (*P* > 0.16), but we observed differences in the HE versus LE cows over time (*P* < 0.001; Figure 1D). The HE-R and HE-NR categories during the treatment phase consumed more feed, respectively, whereas LE-R and LE-NR categories consumed less feed, respectively (*P* < 0.01; Figure 1D). Finally, the EB of HE-R was increased compared with the LE-R cows during the treatment period (*P* < 0.001; Table 1), whereas no differences in EB were detected between HE-NR and LE-NR subgroups (*P* = 0.85; Figure 1, Table 1).

**Figure 1:**
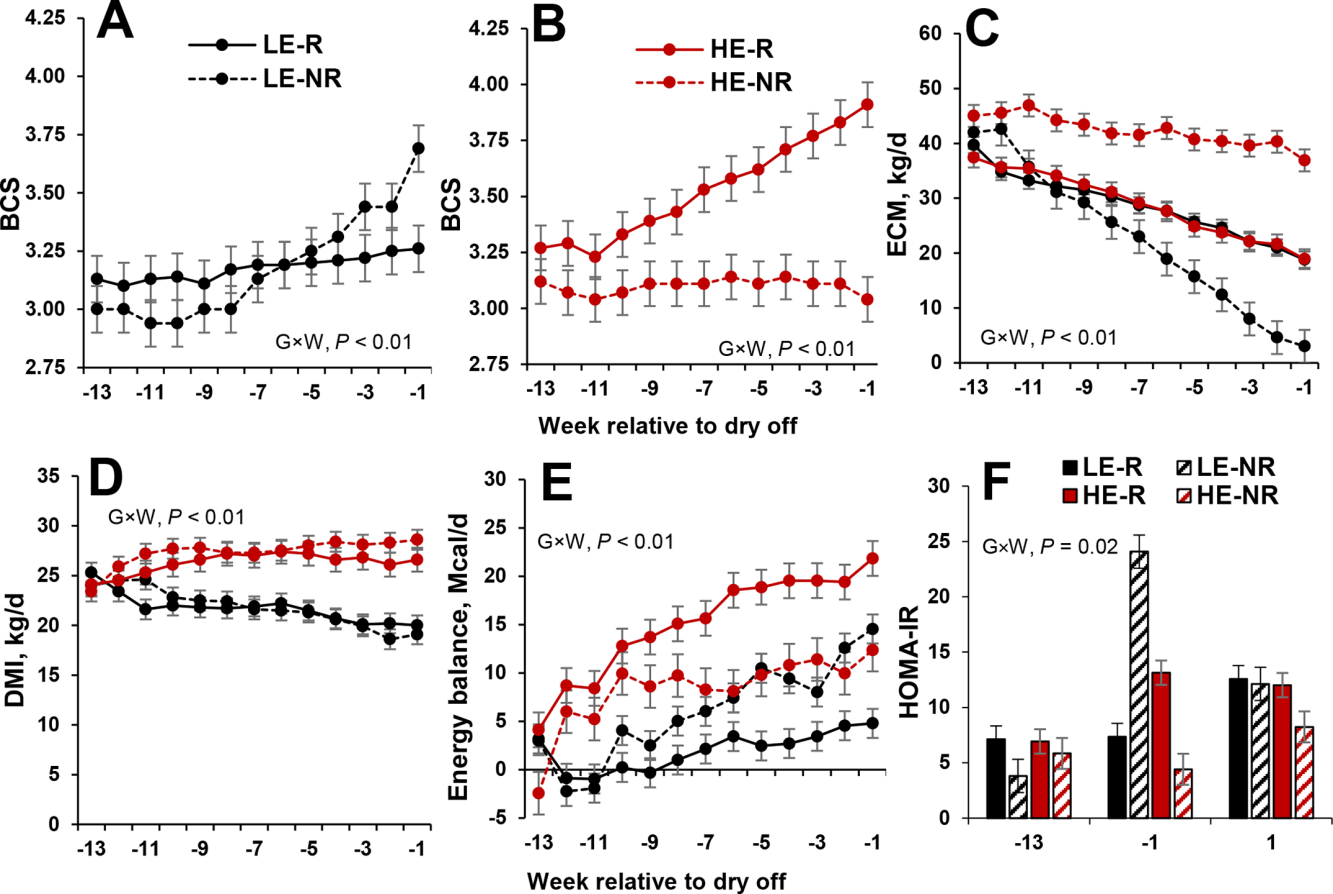
Effect of dietary treatment (LE, HE) from 150 d of gestation (wk −13) to the end of lactation or dry-off (wk −1) on mean (±SEM) BCS (A–B), weekly ECM yield (C), weekly DMI (D), weekly energy balance (E), and calculated HOMA-IR (F; including 1 wk before calving [wk 1]). HE-R = HE responsive; LE-R = LE responsive; HE-NR = HE nonresponsive; LE-NR = LE nonresponsive; G×W = group × week interaction.

**Table 1:** Physiological changes in multiparous cows from the high-energy responsive (>0.25 BCS increase), high-energy nonresponsive (≤0.25 BCS increase), low-energy responsive (≤0.25 BCS increase), and low-energy nonresponsive (>0.25 BCS increase) dietary groups^1^

Item	HE-R (28/35; 80%)	HE-NR (7/35; 20%)	LE-R (27/31; 87%)	LE-NR (4/31; 13%)	*P-*value		
					Time	Treatment	Time × treatment
BFT at dry-off (mm)	45.8 ± 1.7	36.8 ± 2.2	37.9 ± 1.0	41.3 ± 2.2	—	0.01	—
BFT near calving (mm)	45.6 ± 1.8	35.2 ± 1.7	36.3 ± 0.9	37.0 ± 1.5	<0.001	0.01	0.07
Cumulative ECM (kg)	3,304 ± 151	2,236 ± 151	1,622 ± 229	2,608 ± 77	—	0.01	—
ECM before dry-off (kg/d)	18.9 ± 2.2	36.9 ± 2.5	18.8 ± 1.4	3.0 ± 1.4	<0.001	0.0003	0.004
DMI before dry-off (kg DM/d)	20.9 ± 0.8	22.8 ± 1.6	23.5 ± 0.7	21.9 ± 1.4	0.02	0.01	0.13
DMI 21 d pp (kg DM/d)	22.0 ± 0.8	25.2 ± 1.3	24.8 ± 0.7	24.4 ± 1.0	<0.001	0.02	0.66
EB during treatment (Mcal/d)	15.1 ± 1.6	8.29 ± 1.5	1.98 ± 1.6	6.07 ± 3.0	<0.001	0.28	0.43
EB at dry-off (Mcal/d)	21.8 ± 2.3	12.4 ± 1.7	4.79 ± 1.1	14.5 ± 1.5	—	0.01	—
Insulin at dry-off (mU/mL)	17.0 ± 3.0	5.40 ± 1.0	9.80 ± 2.0	23.6 ± 4.5	—	0.001	—
Glucose (mg/dL)	3.54 ± 0.1	3.22 ± 0.2	3.29 ± 0.1	3.33 ± 0.3	0.39	0.07	0.81
HOMA-IR	2.33 ± 0.1	1.79 ± 0.3	2.16 ± 0.1	2.34 ± 0.3	0.004	0.91	0.65

1 All values are reported as LSM ± SEM. HE-R: high-energy responsive; HE-NR = high-energy nonresponsive; LE-R = low-energy responsive; LE-NR = low-energy nonresponsive; BFT = backfat thickness; EB = energy balance; pp = postpartum; HOMA-IR = homeostatic model assessment for insulin resistance.

The HOMA-IR is depicted in Figure 1F and Table 1. At enrollment, HOMA-IR was similar among all treatment groups (*P* = 0.91). However, significant differences were detected at dry-off (*P* = 0.004). We found no significant interaction between treatment and time (*P* = 0.65).

During the first 3 weeks of the dry period, the differences in BCS for HE-R, HE-NR, and LE-R remained constant (*P* > 0.15), whereas the LE-NR subgroup increased BCS from 3.7 to 3.9 ± 0.2 (*P* = 0.002). No changes in BFT were detected during the same period (*P* = 0.29; Table 1).

During the last week of pregnancy, BCS tended to differ between HE-R and HE-NR (*P* = 0.06; 3.84 vs. 3.21 ± 0.2, respectively), whereas LE-R and LE-NR did not (*P* = 0.52; 3.32 vs. 3.87, respectively). Back fat thickness was 43.4 and 39.9 ± 1.1 mm for HE-R and HE-NR, respectively, whereas it was 40.0 and 48.0 ± 1.2 mm for LE-R and LE-NR, respectively. Despite numerical differences, no significant differences were detected between subgroups (*P* = 0.54). We also observed no differences in circulating insulin concentrations among subgroups at 7 d before parturition (*P* = 0.75; Table 1). We did not observe any differences in calf weights due to dietary treatment (*P* = 0.53); however, we observed differences in calf weight between sexes (*P* = 0.002).

Feed intake was similar among subgroups during the first 21 d postpartum, except for the HE-R group, which had a reduction of 2.4 ± 1 kg DM/d (*P* = 0.04; Table 1). Milk yield was similar among subgroups (*P* > 0.45), averaging 50.3 and 51.3 ± 0.6 kg/d during the first 21 and 90 DIM, respectively. No differences in BFT were detected during the first 21 DIM or the entire 90-d period (*P* = 0.64; Table 1). As reported in Cunha et al. (2025), we observed no treatment differences in BHB or NEFA postpartum.

Our primary objective was to investigate the effects of 2 dietary treatments with differing energy densities on body reserves during late lactation and their subsequent impact on the dry period and lactation. Herein, we demonstrated that milk production in the next lactation was not responsive to dietary treatments during the late-lactation period. The unaffected milk yield response in the next lactation appears to be regulated by alterations in insulin concentrations, which were positively associated with the gain of body reserves during the dietary treatment phase, regardless of dietary energy level. It is important to note that this study is exploratory in nature, given that these cows were nonresponsive to nutritional treatments in our original work (Cunha et al., 2025).

Interestingly, it appears that regardless of dietary treatment, milk production was responsible for increasing or maintaining BCS during the dietary treatment period. The homeorhetic adaptation to lactation in dairy cows is a key element in regulating and coordinating energy partitioning, particularly glucose for milk synthesis and lipid metabolism (Bauman and Davis, 1974). Uncoupling the somatotropic axis is a proposed mechanism responsible for producing copious amounts of milk in early lactation (∼21 to 100 DIM) dairy cows. This process favors glucose away from body reserves to the mammary gland for milk production. With the progression of lactation, cows reach positive EB, and the uncoupling of the somatotropic axis is reversed (Sharma et al., 1994; Kobayashi et al., 1999), leading to an increase in body reserves. The nonresponsive subgroups, although numerically small, suggest that some cows have different homeostatic setpoints for energetic regulation during late lactation and eventually later reach the same target of body reserves, based on the changes in insulin concentrations and milk production observed in the nonresponsive and responsive cows fed LE and HE diets. Although the precise mechanisms were not examined in this experiment, alterations in milk production by the mammary gland in response to diet appear to explain the biological variation (Baumgard et al., 2017). This should be explored explicitly in future studies given that this study is exploratory in nature.

Insulin is a potent anabolic hormone that plays a key role in nutrient partitioning to the mammary gland during lactation, particularly during early lactation. Insulin secretion, circulating insulin concentrations, and insulin sensitivity of peripheral tissues are the main determinants of energy partitioning to peripheral tissues or the mammary gland (De Koster and Opsomer, 2013). As part of homeorhetic adaptation in early-lactating dairy cows, insulin resistance occurs, potentially due to increased growth hormone and reduced IGF-1 concentrations, resulting in redirection of glucose to the mammary gland from insulin-dependent peripheral tissues to support milk production (Bell, 1995). This is consistent with observations that cows with higher milk production generally exhibit lower insulin concentrations than those with lower milk production (Lucy, 2019; Zinicola and Bicalho, 2019). This aligns with our observation that cows in the LE-NR group with reduced milk production also had increased HOMA-IR. Additionally, the HE-NR had the lowest HOMA-IR, which is reflective of their increase in milk production in response the HE diet, rather that increasing their body reserves. Further, IGF-1 concentrations are also speculated to influence the amount of milk produced and may also be involved in the adaptation of specific cows to dietary treatment (Capuco et al., 2003; Taylor et al., 2004).

It is unclear why cows converged to the same concentrations of insulin one week before parturition. Generally, in late gestation, pancreatic insulin secretion has been reported to diminish (Rhoads et al., 2004), and tissue sensitivity and responsiveness to insulin action are reduced, essentially leading the cow into an insulin-resistant state (De Koster and Opsomer, 2013). These homeorhetic changes aim to direct an adequate amount of glucose toward the mammary gland, preparing the cow for lactogenesis. Rhoads et al. (2004) suggested that insulin regulates the efficiency of growth hormone signaling in liver and adipose tissue of dairy cows by acting as a rheostat of growth hormone receptor synthesis. Alternatively, the lack of differences in the concentration of circulating insulin 7 d before parturition among subgroups also indicates that cows have reached their target amount of body reserves or that the dry cow diet did not provide adequate glucogenic substrates to drive differences. Although the sample size of our populations and the exploratory nature of this group of cows that were nonresponsive to dietary treatment does not allow us to assess genetics, it has been suggested that the biological drive for a cow to attain and achieve a targeted BCS appears to be as strong as the drive to achieve a genetically programmed peak milk yield (Holmes, 1988; Garnsworthy, 2006). It is possible that this is what occurred in our study; however, this should be further investigated in a prospective experiment with a sufficient sample size.

Our approach of randomly selecting cows at the initiation of our original study resulted in the formation of unexpected subgroups of cows in terms of their response to the dietary treatments. Our study demonstrates that the energy density of their diet influences BCS dynamics and EB patterns in Holstein cows during late lactation and their subsequent lactation and DMI responses. Because this study was performed post hoc and was a secondary observation of the original study, the sample size is small, and future work should include more animals, as well more intensive sampling of these cows, as their physiology appears to be unique. Overall, cows fed a high-energy diet exhibited different BCS and energy dynamics compared with those on a low-energy diet, with notable differences in feed intake and ECM production. Although nutritional management can effectively support the regulation of body reserves, the patterns of fat mobilization and EB are not uniform across all cows as individuals adjust their milk production in response to diets provided as we observed in our nonresponsive cows.
